# Osteoprogenitor cells from non-regenerative bone show greater resistance to cellular stress than those from regenerative bone

**DOI:** 10.3389/fcell.2025.1684670

**Published:** 2025-12-18

**Authors:** Joshua Broussard, Sylvia Culpepper, Tyrel Long, Alexander J. Trostle, Robert J. Tower, Mimi C. Sammarco, Jennifer Simkin

**Affiliations:** 1 Department of Orthopedic Surgery, Louisiana State University Health Sciences Center, New Orleans, LA, United States; 2 Department of Microbiology, Immunology and Molecular Genetics, University of Kentucky, Lexington, KY, United States; 3 Department of Surgery, University of Texas Southwestern Medical Center, Dallas, TX, United States; 4 Department of Orthopedic Surgery, Mayo Clinic, Rochester, MN, United States

**Keywords:** regeneration, bone, periosteal, osteoblast, cellular stress

## Abstract

**Introduction:**

Bone regeneration following injury depends on osteoprogenitor cells derived predominantly from the periosteum. Incomplete regeneration has been attributed to both cell-extrinsic factors (e.g., environment, inflammation, mechanical instability) and cell-intrinsic factors (e.g., impaired proliferation or differentiation of stem cells). In the digit amputation mouse model, amputation through the third phalanx (P3) supports complete regeneration, while amputation through the second phalanx (P2) results in callus formation and scarring. Periosteal cells are known to be the major contributing cell source for repair and regeneration. Yet the healing outcomes of P2 and P3 amputations are significantly different. This study tests whether P2 and P3 cells are functionally equivalent.

**Methods and results:**

Using *in vitro* cellular stress tests, we compared the intrinsic properties of periosteal cells from P2 and P3 bones and found that P3 periosteal cells were more prone to proliferative senescence and less resistant to cellular stress *in vitro* than those from P2. *In vivo*, senescent cells were detected at both P2 and P3 injury sites, but their senescence-associated secretory phenotypes (SASPs) differed depending on the amputation level. Specifically, P2 cells expressed higher levels of pro-inflammatory cytokines (e.g. *Tnf, Il1b*) whereas P3 cells expressed higher levels of protease inhibitors (e.g. *Serpine1, Timp2*).

**Discussion:**

Together, these findings suggest that periosteal cells exhibit intrinsic differences based on anatomical location, which may influence their regenerative capacity and contribute to different healing outcomes.

## Introduction

Bone fractures are a common clinical problem, with approximately 178 million new cases reported worldwide in 2019 (Wu, 2021). Successful fracture repair depends on the proliferation and differentiation of osteoprogenitor cells from the periosteum to replace bone. Disruption of this process can result in nonunion healing, where bone regeneration fails to proceed effectively (Wang et al., 2017; Colnot, 2009). Although most fractures heal without complication, 5%–10% fail to do so, resulting in nonunion, malunion, osteomyelitis, or chronic pain, often necessitating invasive surgical intervention (Rupp et al., 2018; Tzioupis and Giannoudis, 2007; Nicholson et al., 2021).

The etiology of nonunion healing is multifactorial. Known risk factors include infection, comorbidities such as diabetes or smoking, advanced age, and mechanical instability at the fracture site (Saul et al., 2023; Wildemann et al., 2021). However, many patients without these risk factors still experience impaired healing and remain unresponsive to current therapies (Nicholson et al., 2021). Current treatments such as bone morphogenetic proteins (BMPs), platelet-derived growth factor (PDGF), or low-intensity ultrasound are designed to stimulate osteogenesis by enhancing local osteoprogenitor activity (Alkindi et al., 2021; Nevins et al., 2003; Nevins et al., 2013; Böhm et al., 2019; Salazar et al., 2016; Khan and Laurencin, 2008). Yet, studies isolating osteoprogenitor cells from nonunion fractures show that these cells exhibit reduced osteogenic differentiation and increased cellular senescence compared to bone marrow derived stromal cells from uninjured sites (Bajada et al., 2009), suggesting that intrinsic defects within the cells themselves may limit their responsiveness to pro-osteogenic therapies.

Interest in mesenchymal stem cell (MSC) therapies has grown due to their accessibility and expansion potential, particularly bone marrow- and adipose-derived stem cells (Littman and Abo, 2015; Jin and Lee, 2018; Mousaei Ghasroldasht et al., 2019; Watson et al., 2014). However, periosteum-derived MSCs stand out for their robust osteogenic capacity and central role in natural bone repair (Wang et al., 2017; De Bari et al., 2006; Mahardawi et al., 2023; Doi and Sakai, 1994; Nakahara et al., 1990). Although clinical harvest is limited by donor site morbidity, these cells consistently outperform other MSCs in osteodifferentiation, osteointegration, and purity, and engineering periosteal-like grafts may further expand their utility (Ferretti and Mattioli-Belmonte, 2014; Duchamp de Lageneste et al., 2018; Stich et al., 2017). Notably, periosteal cells from different bones exhibit distinct osteogenic profiles, suggesting that regenerative potential may depend on the intrinsic properties of their site of origin (Bilkay et al., 2008).

One cellular mechanism increasingly implicated in impaired bone healing is cellular senescence. Senescent cells, marked by expression of genes such as p16 and p21, senescence-associated secretory phenotypes (SASPs), and β-galactosidase accumulation at pH 6, accumulate at fracture sites and impair healing. Experimental clearance of these cells has been shown to accelerate fracture repair (Saul et al., 2021; Saul et al., 2024; Liu et al., 2022). Moreover, bone marrow derived stromal cells (BMSCs) isolated from atrophic nonunion sites exhibit hallmarks of senescence, including reduced viability, prolonged doubling times, impaired mineralization, and secretion of osteogenesis inhibitors (Bajada et al., 2009). These findings suggest that senescence, whether induced by aging or cellular stress, may directly compromise the reparative capacity of osteoprogenitor cells.

However, the relationship between senescence and regeneration is not straightforward. In some regenerative mammals, such as the African spiny mouse, enhanced resilience to stress-induced senescence correlates with superior regenerative capacity compared to non-regenerative rodents (Saxena et al., 2019). This has led to the hypothesis that resistance to senescence may promote regeneration. Yet, in contrast, studies on highly regenerative vertebrates such as salamanders and zebrafish suggest that senescent cells can transiently accumulate to actively promote tissue remodeling and regeneration (Yun et al., 2015; Yun et al., 2013; Feng et al., 2019; Silva-Álvarez et al., 2019), implying that senescence can serve both beneficial and detrimental roles depending on context, tissue type, or species.

These contrasting observations raise a key question in regenerative biology: how do tissue-resident progenitor cells regulate senescence during injury repair, and does this regulation differ between regenerative and non-regenerative tissues?

To address this question, we examine periosteal MSCs from two distinct long bone locations in mice that differ in regenerative capacity. In the mouse digit amputation model, the distal third phalangeal element (P3) undergoes complete bone and tissue regeneration following amputation, whereas the proximal second phalangeal element (P2) heals with scar formation and incomplete bone regeneration (Simkin et al., 2013; Borgens, 1982; Fernando et al., 2011; Neufeld and Zhao, 1995). Based on the regenerative ability of P3, we initially hypothesized that P3 periosteal cells would exhibit greater resistance to cellular stress and senescence than P2 cells. Surprisingly, our findings reveal the opposite: P3 cells accumulate senescence markers more readily than P2 cells, display reduced proliferation, and are more sensitive to oxidative stress. These data suggest that senescence may serve distinct functions during regenerative versus scarring responses and highlight the need to better understand how senescence pathways are differentially engaged depending on the regenerative context.

## Results

### P3 periosteal cells have decreased proliferative capacity and increased proliferation-induced senescence

**FIGURE 1:**
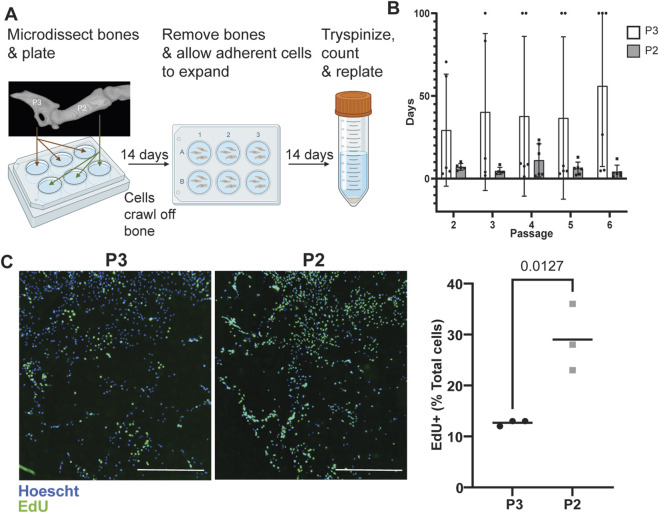
P2 periosteal cells display greater proliferative capacity *in vitro.* **(A)** Schematic of P2 and P3 periosteal cell isolation. Made with BioRender. **(B)** Doubling time (max 100 days) was calculated at each passage. n = 6 animals, mixed-effects analysis, main effect passage number (F = 0.41, p = 0.6); main effect cell origin (F = 5.19, p = 0.04). Data are shown as mean ± standard deviation. **(C)** Quantification of EdU-positive cells as percent of total cells in well and representative images of EdU staining for P3 and P2 cells at passages 3 and 4. n = 3 samples/group, Scale bar = 1 mm EdU staining (green), nuclear staining (blue, Hoechst). Data are shown as mean.

After P3 digit amputation, periosteal cells proliferate and differentiate to form the new periosteum and much of the newly regenerated bone (Johnson et al., 2020; Rinkevich et al., 2011; Lehoczky et al., 2011; Dawson et al., 2018). In P2, these same periosteal cells proliferate to form a callus and scar (Dawson et al., 2016). We first sought to test inherent differences in proliferation between progenitor osteoblasts from the periosteum of uninjured P2 or P3 bones. To do this, we isolated and cultured P3 and P2 periosteal cells from adult (8-week-old) CD1 outbred mice (Figure 1A) (Busse et al., 2019). We observed a significantly faster doubling time for P2 (6.70 days, n = 6 mice) vs. P3 cells (39.57 days, n = 6 mice), independent of passage number (mixed-effects analysis with main effects passage number F = 0.41, p = 0.63 and cell type F = 5.19, p = 0.04). This P2 doubling time remained consistent in all six mice across time (Figure 1B). In contrast, P3 cells showed high variability across animals. Although three of the six samples demonstrated similar doubling times to P2 cells (6.70 days), two of the six samples were considerably slower and displayed doubling times of >50 days in culture from the onset. Furthermore, three of the six samples completely stopped doubling by passage 5 (Figure 1B). To test proliferation ability *in vitro* more accurately, we performed EdU staining at passage 3. P2 cells showed more EdU-positive cells than P3 cells (29% vs. 12.7%, unpaired t-test, p < 0.05, n = 3 mice Figure 1C).

**FIGURE 2:**
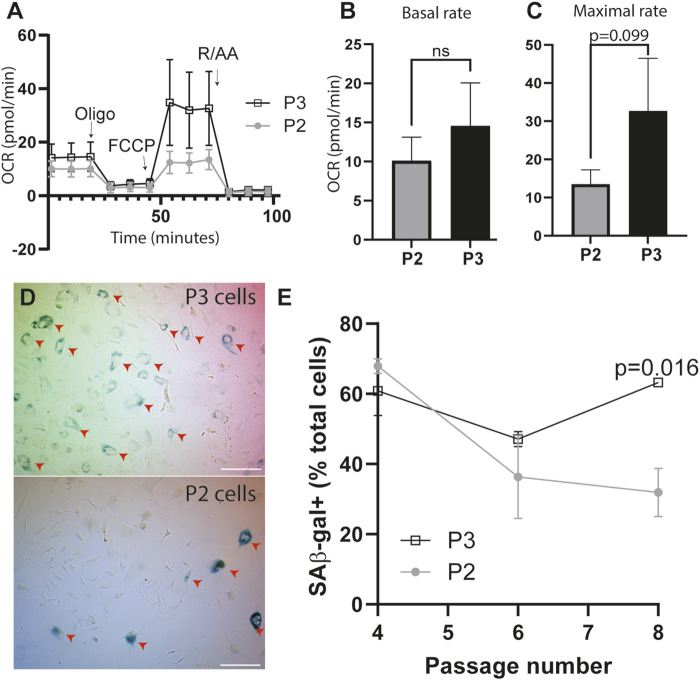
P3 cells display increased oxidative metabolism and increased lysosomal content compared to P2 cells *in vitro.* **(A)** Seahorse MitoStress Test Analysis to measure the oxygen consumption rate (OCR) of P2 and P3 periosteal cells graphed over time. Oligo, oligomycin; FCCP, R/AA, rotenone/antimycin. **(B)** P3 and P2 cell basal OCR. **(C)** P3 and P2 maximal respiratory rate. T-test with Welch’s correction, n = 10 technical replicates, Data are shown as mean ± standard deviation, p ≤ 0.05. **(D)** Representative images from SA-βGal senescent staining of P3 (top) and P2 (bottom) cells at passage 8. Red arrows represent the SA-βGal-positive cells. Scale bar—50 μm. **(E)** Quantification of A showing number of SA-βGal-positive cells as a percent of total cells during passaging of P2 and P3 periosteal cells. n = 3 animals, Sidak’s multiple comparison test p = 0.016. Data are shown as mean ± standard deviation.

Because P3 cells stop dividing *in vitro*, we measured metabolic capacity of these cells to identify a potential explanation for loss of proliferation. Using the Agilent Seahorse MitoStress Test, we measure similar basal rates of oxidative metabolism between P2 and P3 cells (unpaired t-test with Welch’s correction, p = 0.08, n = 10 technical replicates, Figures 2A,B). However, P3 cells demonstrate a significantly greater maximal respiratory capacity than P2 cells (unpaired t-test with Welch’s correction, p = 0.01, n = 10 technical replicates Figures 2A,C). Thus, despite decreased proliferative capacity observed in P3 cells, we note an increase in oxidative metabolism in these cells, indicative of potential cellular senescence (Suryadevara et al., 2024).

Cellular senescence is typically defined by cell cycle arrest (Khosla et al., 2022). With regard to regeneration, senescence data have been conflicting. Senescence is a critical component in regeneration in lower vertebrate models (Yun et al., 2013; Silva-Álvarez et al., 2019). Conversely, regenerative cells can resist cellular senescence via alternative regulation of tumor suppressor pathways compared to non-regenerative counterparts (Gawriluk et al., 2016; Brookes et al., 2004). To determine whether decreased proliferation alongside increases in oxidative metabolism in P3 periosteal cells is indicative of increased senescence, we quantified senescence using SA-β Gal, which measures changes in lysosomal content often associated with senescence (Figure 2D) (Saxena et al., 2019). SA-β Gal studies show that P2 and P3 periosteal cells initially have the same starting populations of SA-β Gal-positive cells at passage 4. Although this P2 SA-β Gal-positive population decreases with subsequent cell passages (54% decrease), the percentage of P3 SA-β Gal-positive cells remains high during passaging, with an average 3.6% increase by passage 8 (Figure 2E, n = 3 mice, two-way ANOVA with main effects cell type and passage number, followed by Sidak’s multiple comparison test, *p < 0.05). Together, these data demonstrate that P3 periosteal cells maintain a steady SA-β Gal-positive cell population through passaging, compared to P2, and that this also corresponds to a shorter replicative lifespan, longer doubling times, and higher oxidative metabolism rates, consistent with the characteristics of senescent cells (Suryadevara et al., 2024).

### P3 periosteal cells are more susceptible to ROS-induced senescence

**FIGURE 3:**
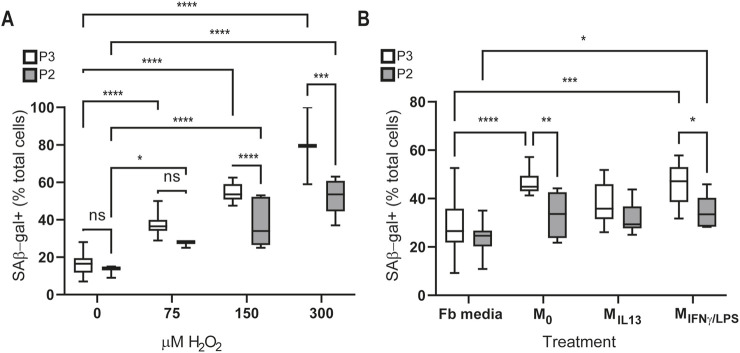
P2 cells are more resistant to stress *in vitro* than P3 cells. **(A)** SA-βGal assay showing the number of P2 and P3 senescent cells as percent of total cells after treatment with increasing concentrations of H_2_O_2_ (two-way ANOVA with main effects concentration of H_2_O_2_ and cell type, n = 3–6 technical replicates per group). **(B)** SA-βGal assay comparing senescence in P2 and P3 cells exposed to macrophage-conditioned media. Control group, P2/P3 cells were exposed to DMEM+20%FBS (Fb media). M_0_ group, P2/P3 cells were exposed to BMDM macrophage media with no stimulation. M_IL13_ group, P2/P3 cells were exposed to BMDM macrophage media with IL13 stimulation. M_IFNγ/LPS_ group, P2/P3 cells were exposed to BMDM macrophage media with IFNγ/LPS. n = 6 technical replicates. *Post hoc* Fisher’s LSD *p < 0.05, **p < 0.01, ***p < 0.001, and ****p < 0.0001. Data are shown as mean with whiskers indicating the minimum to maximum values.

Injury can lead to increased reactive oxygen species (ROS) production, which, in turn, can trigger senescence (van der Vliet et al., 2014). Resistance to ROS-induced senescence has been associated with increased proliferative ability (Saxena et al., 2019; Bilgen et al., 2019). To test whether the decreased proliferative capacity and increased senescence of P3 periosteal cells are associated with decreased ROS resistance, we treated P2 and P3 periosteal cells with H_2_O_2_ and measured the effects on senescence, as defined by SA-β Gal staining. Treatment with H_2_O_2_ resulted in increased numbers of SA-β Gal-positive cells in both P2 and P3 cell populations (Figure 3A) (two-way ANOVA main effect H_2_O_2_ concentration F = 59.65, p < 0.0001). However, increasing concentrations of H_2_O_2_ resulted in a 5-fold increase in SA-β Gal-positive P3 cells compared to a 4-fold increase in P2 cells, indicating a greater H_2_O_2_-induced transition to senescence in P3 periosteal cells (Figure 3A 2-way ANOVA main effect cell type, F = 30.18 p < 0.0001). Although both P2 and P3 cells show significant increases in SA-β Gal-positive cells with increasing concentrations of H_2_O_2_, P3 cells demonstrate a significantly higher percent of SA-β Gal-positive cells than P2 at H_2_O_2_ concentrations ≥150 μM (54.29% vs. 37.5%, 150 μM and 79.5% vs. 52.3%, 300 μM, Fisher’s LSD *post hoc*, p < 0.001) (Figure 3A). Combining these data suggest that P3 has a limited capacity to withstand ROS-induced increases in lysosomal content often associated with senescence.

### P3 periosteal cells are more susceptible to M0 and M_IFN/LPS_ macrophage-induced senescence

After bone injury, including digit tip amputation, macrophages are among the first cells to respond to the site and shift the environment. Changes in the macrophage secretome are linked to poor fracture healing and increased senescence, especially in the context of aging or chronic inflammatory conditions (Clark et al., 2020; Hou et al., 2024). Additionally, inflammatory macrophages induce senescence in fibroblasts, both *in vivo* and *in vitro*, through secretory factors such as TNFα and ROS (Sindrilaru et al., 2011; Li et al., 2017). Given that P3 periosteal cells exhibited increased senescence in response to ROS, we investigated the impact of macrophage-conditioned media. We treated bone marrow macrophages with IFNγ/LPS (M_IFNγ/LPS_), IL-13 (M_IL-13_), or no treatment (M_0_) for 24 h to generate macrophage-conditioned media (MCM) and cultured P2 and P3 periosteal cells for 24 h in MCM before assaying for SA-βGal. Although P2 cells showed no significant change in SA-βGal in any treatment group (two-way ANOVA: F = 1.094, p = 0.3583), P3 cells showed a significant increase in SA-β Gal-positive cells when exposed to M_0_-conditioned media (18.7%, P=<0.0001) and M_IFNγ/LPS_-conditioned media (18.3%, P = 0.0001) (Figure 3B). These data suggest that P3 periosteal cells are more primed to initiate increases in lysosomal content often associated with senescence in response to macrophage signals after injury than P2 cells.

### Senescence is an inherent part of regeneration and scar formation

**FIGURE 4:**
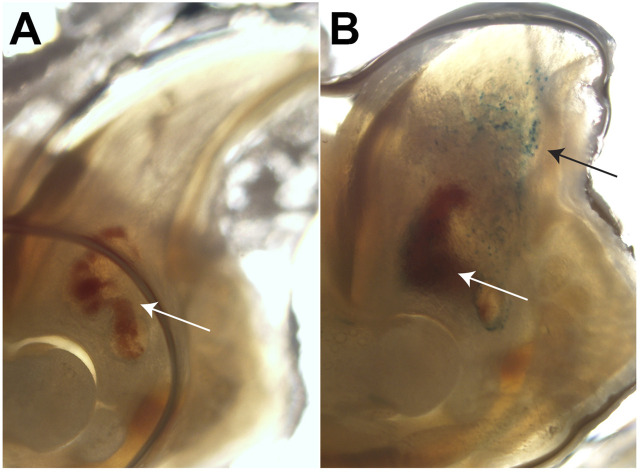
P3 digits 10 days after injury show senescent cells within the blastema region. **(A)** SA-βGal staining (pH 6) of an unamputated P3 and **(B)** a day-10 blastema in slice culture. White arrows indicate the marrow cavity, and black arrows indicate SA-βGal-positive cells within the blastema.

We next sought to determine whether P3 senescence is evident during regeneration. The SA-βGal assay that is used to identify senescence is cell-type-dependent and measures the increase in lysosomal mass observed in senescent cells (Dimri et al., 1995; Kurz et al., 2000; van der Loo et al., 1998). In this assay, cells are stained with the chromogenic substrate X-gal at either pH 4 or pH 6. Staining at pH 4 serves as a positive control and should stain the majority of cells blue, whereas staining at pH 6, a suboptimal pH for X-gal, can only detect those cells with increased lysosomal mass. To address the multi-tissue environment of the regenerating P3, we utilized a slice culture model developed by our team previously (Sammarco et al., 2014) and investigated SA-βGal staining during blastema formation at day 10. Control staining at pH 4 shows that most tissue, including the marrow, in P3 stains blue as a positive control, as expected (Supplementary Figure S1). However, staining at pH 6 at DPA 10 shows SA-βGal-specific cells in the blastema. This SA-βGal staining is in comparison to the unamputated digit, which shows very little SA-βGal signal at pH 6 (Figures 4A,B). These data parallel data from other regenerative models (Yun et al., 2013; Feng et al., 2019; Silva-Álvarez et al., 2019; Demaria et al., 2014), suggesting that senescence may be an inherent and potentially integral part of regeneration. Further staining with the cell cycle inhibitor p21(Waf1/Cip) showed scattered p21^+^ cells within the P3 blastema at day 10 and in the scar-forming region next to the bone of P2 digits (Supplementary Figure S2), indicating the presence of cells in cell cycle arrest in both injuries.

**FIGURE 5:**
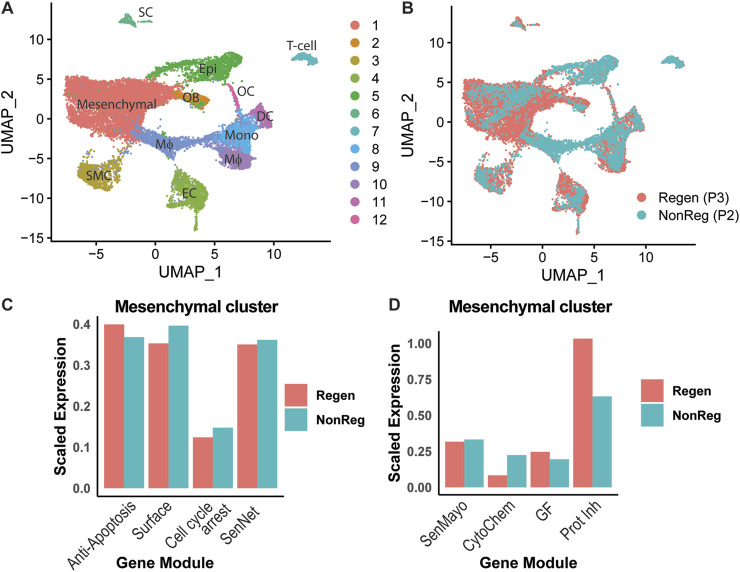
P2- and P3-derived cells express differential senescent phenotypes. **(A)** UMAP for single-cell RNAseq data from P2 and P3 amputated digits at 10 and 14 days post-amputation defining clusters by cell type. **(B)** UMAP defining P2 (NonReg) and P3 (Regen) clusters by injury type. **(C)** Modular scores for SenNet gene expression are quantified to compare P2 and P3 injuries across cluster 1 (mesenchymal cells). **(D)** Modular scores for SenMayo gene expression are quantified to compare P2 and P3 injuries in cluster 1 (mesenchymal cells). OB, osteoblast; OC, osteoclast; DC, dendritic cell; MΦ, macrophage; Mono, monocyte; SC, Schwann cell; SMC, smooth muscle cell; EC, endothelial cell; Epi, epithelial cell. SenNet [modular score of senescent genes common to most tissues combined (Suryadevara et al., 2024), SenMayo (modular score of senescent genes found in bone combined (Saul et al., 2022)]. CytoChem, cytokine and chemokine module; GF, growth factor module; Prot Inh, protease inhibitor module.

However, because cellular senescence is difficult to determine based on a single marker (Suryadevara et al., 2024), to further investigate the levels of senescent cells during regeneration versus fibrosis, we leveraged two published single-cell RNAseq datasets that isolated cells from the injury site of P2 and P3 at days 10, 11, and 14 after injury (Johnson et al., 2020; Storer et al., 2020). Unsupervised clustering identified 12 unique clusters, including two groups of mesenchymal lineage cells (mesenchymal and osteoblasts), six groups of hematopoietic cells (macrophages, monocytes, dendritic cells, osteoclasts, and T-cells), and one group each of endothelial cells, smooth muscle cells, Schwann cells, and epithelial cells (Figure 5A). All groups were present in both P2 and P3 injury sites (Figure 5B).

To compare cellular senescence in these datasets, we used two recent panels of genes recommended as potential senescent markers. The first panel, dubbed SenNet, contains 37 genes identified as common senescent markers across tissues (Suryadevara et al., 2024), whereas the second panel, dubbed SenMayo, contains 125 genes specific to senescent cells in bone (Saul et al., 2022). These panels confirmed our slice culture findings that senescent cells are present within the mesenchymal population (cluster 1) of regenerative injuries but also highlighted that senescent cells are present in the mesenchymal population of fibrotic injuries as well (Figures 5C,D SenNet and SenMayo). Direct comparison of P2 fibrotic cells to P3 regenerative cells shows that the combined SenNet profile is slightly higher in P2 during cartilaginous callus formation than in P3 blastema formation when comparing at all cells of the digit (Supplementary Figure S3A), whereas the combined SenNet genes are expressed at relatively equal levels within the mesenchymal cluster alone (Figure 5C, SenNet). In comparison, the SenMayo gene panels are expressed at relatively equal levels in all cells and in the mesenchymal cluster alone during P2 cartilaginous callus formation and P3 blastema formation (Supplementary Figure S3B, Figure 5D, SenMayo). To further investigate the two senescence profiles, we segmented the gene panels into groups according to function and applied these profiles again to the P2 callus formation stage and P3 blastema stage (Supplementary Figure S3). We found that scar-forming injuries were more likely to exhibit high expression of the cytokine and chemokine genes (e.g., *Ccl1*, *Ccl4*, *Il13*, *Il18*, *Il1a*, *Il1b*, *Il7*, *Spp1*, and *Tnf*; full panel Supplementary Table S1) in the SenMayo SASP profile compared to P3 cells (Figure 5D, CytoChem). In contrast, P3 cells were more likely to express the protease inhibitors (e.g., *Csst10*, *Igfbp1/2/3*, *Serpinb3a*, *Serpine1*, and *Timp2*; full panel Supplementary Table S1) (Figure 5D, Prot Inh). Together, these data suggest that, in both regenerating and scar-forming injuries, senescent cells are present, but the profiles of senescent genes expressed are unique to the injury site.

## Discussion

Periosteal cells are the primary contributors to healing in both P2 and P3 amputation models, yet these injuries result in markedly different outcomes (Simkin et al., 2013; Dawson et al., 2018; Dawson et al., 2016). These divergent responses may stem from either extrinsic environmental differences or intrinsic properties of the periosteal cells themselves. In this study, we focused on testing the latter. Our findings reveal intrinsic differences in P2 and P3 periosteal cells, including divergent responses to stress, varying levels of senescence, and distinct proliferative capacities *ex vivo*. Prior studies suggest that endosteal cells also contribute to digit regeneration, although to a lesser extent than periosteal cells (Dawson et al., 2018). Our approach may be limited by the fact that our *in vitro* cell populations may contain ratiometrically different endosteal and periosteal cell populations from those found *in vivo*, given the approach to allow periosteal cells to migrate off the bone. As such, we cannot fully scrutinize the contribution of endosteal cells to differences in senescence.

Traditionally, cell cycle arrest has been considered a defining feature of senescence (Khosla et al., 2022). However, recent work by Saul et al. challenges this view, describing a proliferative osteochondro-progenitor population that also exhibits a senescent phenotype during fracture healing. This work broadens the definition of senescence and suggests that senescent states can differ based on the context, such as injury versus aging (Saul et al., 2024). This variability in senescence also raises questions about how we interpret proliferation during regeneration, particularly given that commonly used markers may not capture the full complexity of cell state. Proliferation in regenerative tissue is often assessed *in situ* using markers including PCNA and Ki67, rather than direct functional assays. Our prior work showed that although P3 exhibits more proliferation than P2, the proliferating population is relatively small, consistent with the idea that regeneration depends on lineage-restricted dedifferentiation rather than the generation of new cells (Johnson et al., 2020; Rinkevich et al., 2011; Sammarco et al., 2014). Curiously, when cultured *ex vivo*, P2 periosteal cells maintain proliferative activity longer than P3 cells and are associated with lower senescence, paralleling results observed in fracture repair (Saul et al., 2024). In contrast, P3 cells show greater variability in doubling time and a progressive decrease in proliferative capacity across passages. Although proliferative decrease is typically representative of aged or stressed tissues, this initial variability may reflect the intrinsic heterogeneity of the periosteal cell population or, more interestingly, represent subpopulations of periosteal cells, highlighting the need for more studies on senescence in regeneration and characteristics of progenitor cell populations.

Interestingly, this intrinsic limitation in P3 cell proliferation appears to shift in aged animals. In older mice, P3 cells exhibit increased proliferation but reduced ability to form patterned bone (Tower et al., 2022). Although increased proliferation is associated with regenerative capacity in other strains such as LG/J (Qu et al., 2025), our findings suggest that proliferation alone is not predictive of regenerative success. Similar observations have been made in other species: regenerative mammals including rabbits and African spiny mice regenerate ear tissue more effectively than rats, yet all three species exhibit comparable proliferative capacity *in vitro* (Saxena et al., 2019; Gawriluk et al., 2016). These comparisons highlight that intrinsic proliferative potential, independent of extrinsic factors, does not necessarily determine regenerative outcomes. Notably, our direct comparison of P2 and P3 cells from the same species and injury model confirms that P3 cells, although isolated from a regenerative injury, possess a more limited proliferative potential, coupled with greater susceptibility to senescence *ex vivo*.

Senescence is also known to be induced by environmental stressors. We tested cell response to stressors by exposing P2 and P3 periosteal cells to H_2_O_2_ and macrophage-conditioned media. Both cell types showed increased senescence in response to H_2_O_2_, but P3 cells were more sensitive, exhibiting senescence at lower H_2_O_2_ concentrations. Similarly, P3 cells responded to both inflammatory (M_IFNγ/LPS_) and unstimulated (M_0_) macrophage media with increased senescence, whereas P2 cells responded only to the inflammatory condition. These results suggest that P3 cells are more prone to entering senescence in response to cues from infiltrating immune cells. As expected, neither cell type responded to anti-inflammatory (M_IL13_) media. We, and others, have previously shown that macrophages are required for successful regeneration in the digit and other regenerative models (Simkin et al., 2017a; Simkin et al., 2017b; Simkin et al., 2024; Gawriluk et al., 2020; Godwin et al., 2013; Petrie et al., 2014; Aurora et al., 2014). In particular, macrophages play a dual role in bone repair, initially clearing debris as inflammatory macrophages and later promoting repair as anti-inflammatory macrophages (Einhorn and Gerstenfeld, 2015; Alexander et al., 2011; Kaur et al., 2017). Our data suggest that although early inflammation can trigger senescence in both P2 and P3 fibroblasts, P3 cells are intrinsically more vulnerable to this response. Interestingly, although macrophages are essential for regeneration, a common thread across regenerative species is a less robust, shorter burst of initial inflammatory cytokines than species and tissues that fail to regenerate (Gawriluk et al., 2020; Brant et al., 2016; Okamura et al., 2021; Lai et al., 2017). It is possible that intrinsic vulnerability to inflammatory macrophages may help control the extent of the inflammatory response. Further exploration of the *in vivo* profiles of P2 and P3 senescent cells also supports this idea. We analyzed previously published scRNAseq datasets and compared the senescent profiles of mesenchymal cells in P2 and P3. We found that senescence in P2 callus cells was associated with a chemokine-rich SASP (CytoChem), whereas P3 blastema cells displayed a SASP profile dominated by protease inhibitors (Prot Inh). These SASP differences likely influence the local immune and repair environment. Cytokine- and chemokine-rich SASPs are often associated with prolonged inflammation and fibrosis (Bhatt et al., 2025; Wang et al., 2021; Liu et al., 2021), triggering feedback loops that may explain extended inflammatory profiles in non-regenerative tissues. In contrast, profiles rich in ECM remodelers and growth factors (e.g., Mmps and Pdgf-aa) support resolution of injury and regeneration (Demaria et al., 2014), suggesting that unique SASP profiles may be important in determining the healing outcome.

Unexpectedly, we observed an upregulation of protease inhibitors such as Serpine1 in P3 compared to P2. This finding contrasts with studies where increased protease activity has been linked to fibrosis in skeletal muscle and lung (Jiang et al., 2017; Krause et al., 2011). However, context and timing are critical: a transient chemokine SASP may help coordinate early repair, whereas a chronic chemokine response may hinder regeneration (Saul et al., 2021; Saul et al., 2024; Aghali et al., 2022). Our data suggest that early inflammation induces a chemokine-rich SASP in P2 fibroblasts, potentially sustaining fibrosis, whereas in P3, senescent cells promote tissue remodeling through protease inhibitors. The subcategory differences and temporal regulation of senescence further support the idea that P2 and P3 fibroblasts are intrinsically programmed to respond differently to injury and inflammation.

This raises an important question: what role does senescence play in regeneration, and can we manipulate senescent profiles to improve outcomes? Although senolytic treatments have been explored to enhance fracture healing, studies on highly regenerative species such as axolotls and zebrafish suggest that senescence can be beneficial (Yun et al., 2013; Feng et al., 2019; Silva-Álvarez et al., 2019). These models show that senescent cells may support regeneration rather than inhibit it. Interestingly, in mammals, *in vitro* resistance to senescence is observed in both regenerative and non-regenerative species (i.e., rats and rabbits), whereas mouse cells are highly sensitive to stress-induced senescence (Saxena et al., 2019). This suggests that resistance to senescence may not correlate directly with regenerative ability across species. Instead, the type of senescent response and the SASP profile may be more informative. In our model, P3 senescent cells may contribute to regeneration through a remodeling SASP, whereas P2 cells may promote fibrosis via a pro-inflammatory SASP. This hypothesis warrants further investigation.

Altogether, our findings challenge the assumption that periosteal cells from different bones behave uniformly. Instead, we demonstrate that P2 and P3 cells differ intrinsically in their proliferative capacity, stress response, and senescent profiles. These differences may play a key role in determining regenerative versus fibrotic healing outcomes.

## Materials and methods

### Animal care

All experiments were performed in accordance with the standard operating procedures approved by the Institutional Animal Care and Use Committee of LSU Health Sciences Center (Protocol #4754) and the University of Kentucky (Protocol #2024-4471).

### P2/P3 cell isolation, culture, and proliferation assays

To isolate P2 and P3 cells, we followed previously published protocols (Busse et al., 2019). In brief, P3 and P2 bones were micro-dissected from the hind limbs of 8-week-old CD1 mice (Charles River #022), and all ligaments, dermal tissues, and epidermal tissues were mechanically removed. Six digits from the hind limb of a single mouse (digits 2, 3, and 4) were micro-dissected. P3 bones from these digits were placed in a single well of a six-well plate for culture. P2 bones from these digits were placed in a separate well for culture. This process was repeated for n = 3 mice and n = 18 total digits (6 digits per mouse). Cells were cultured with 5 mL of media (high-glucose DMEM, 20% FBS, and 1% PenStrep) and given 2 weeks to mobilize from the bone to the culture plate. The bones were removed from culture after 2 weeks. Cells were dissociated from the plate using 0.25% trypsin–EDTA for 15 min, counted, and transferred to new dishes for passage 1. A total of 1 × 10^5^ cells were replated into a new dish every 7 days. The total cell count was recorded for doubling time calculations and graphed using population doublings (PDs) = ([t *(ln2)]/[ln(Xe/Xb)] (t = days past cell seeding, Xe = number of cells seeded, and Xb = number of cells harvested).

EdU staining was carried out at passage 3 according to the kit manufacturer’s instructions (Invitrogen, 10637). Cells were incubated with EdU for 24 h prior to Click-It staining and visualization on a Cytation 5 (Agilent BioTek). Quantification was carried out in Gen5 software to count EdU-positive (green) cells divided by total (Hoechst-positive) cells.

### Cell culture senescence-associated β-galactosidase (SA-βgal) assay

Cells were seeded onto a 6-well plate and fixed using 1 mL of β-galactosidase staining fixative (Cell Signaling, 9860S) for 10–15 min at room temperature. Fixative was removed, and cells were washed twice with 1X PBS. Cells were stained using 1 mL of the β-galactosidase staining solution at 37 °C overnight in a dry incubator (no humidity or CO_2_). β-Galactosidase activity was confirmed using microscopy. To quantify SA-βGal activity, each well was imaged (10 images at ×200 magnification per well), and blue-stained cells were counted (Cell Count, ImageJ). The procedure was repeated at passages 4, 6, and 8.

### Digit slice SA-βgal assays

For amputations, 8-week-old CD1 (Charles River #022) mice were anesthetized with 3% isoflurane and maintained under anesthesia at 1% isoflurane; for the procedure, hind digits 2 and 4 were amputated at the P3 level, and digit 3 was retained as an unamputated control (Simkin et al., 2013). To identify senescence, we used a protocol by Debacq-Chainiaux et al. (2009) and adapted it to accommodate our digit slice model (Sammarco et al., 2014). In brief, regenerating and unamputated mouse digits were harvested at selected days during the regenerative process. A Leica Vibratome at 1 mm amplitude and 0.1 mm/s progression was used to create a 250-µm slice. The slice was fixed in Z-fix and incubated in either the control (pH 4) or the SA-βGal (pH 6) staining solution overnight at 37 °C and photographed.

### H_2_O_2_ stress assay

Cells were incubated in 6-well plates with serial dilutions of H_2_O_2_ in complete media (high-glucose DMEM with 20% FBS and 1% PenStrep) for 2 h. Following the incubation, cells were washed with PBS to remove residual H_2_O_2_. Fresh medium (1 mL) was added to each well, and cells were incubated for 24 h prior to SA-βGal staining and imaging to assess P3 and P2 cell response to H_2_O_2_-induced stress.

### Inflammatory stress assay

To measure inflammatory stress resistance, P3 and P2 cells were placed in 24-well plates with different variations of MCM. To prepare MCM, mouse bone marrow macrophages were isolated and expanded according to our previously published protocol (Simkin et al., 2017a). Bone marrow macrophages were treated with different stimulants, IFNγ (20 ng/mL, PeproTech 315-05) + LPS (100 ng/mL, from *E. coli* strain O111:B4, Sigma L5293), IL-13 (20 ng/mL, PeproTech 210-13), or no treatment (M0) for 24 h. MCM media were then collected at 24 h and filtered to remove cells. P3 and P2 cells were then cultured in MCM and collected 24 h later for SA-βGal senescent staining.

### Immunofluorescence

Digits were collected at D10 after amputation and fixed in neutral buffered formalin for 24 h. Tissue was decalcified using Decal 1 (Leica, 3800440) for 48 h, washed with PBS, and stored in 70% ethanol until paraffin processing and embedding. Four-micrometer sections were collected for staining. For staining, tissue was deparaffinized and incubated in heat-retrieval solution (pH 6) overnight at 60 °C. To block nonspecific binding, slides were incubated in serum-free blocking buffer (ScyTek AAA999). Staining was carried out at 4 °C overnight using p21 Waf1/Cip1 (clone HUGO-291, Sigma Cat# MABE1816, 1:50 dilution). Slides were washed with PBST and incubated at room temperature for 45 min with the secondary antibody at 1:800 dilution in the serum-free block: donkey anti rat AF488 (Invitrogen, Cat#A21208). Nuclei were counterstained with DAPI (Thermo Fisher, 3375), and slides were coverslipped with ProLong Gold antifade reagent (Invitrogen, Cat# P36930). Images were collected on an Olympus ix85 deconvolution microscope at ×200. A red filter was used to capture background noise. Representative images were shown from n = 3 animals.

### Seahorse analysis

P3 and P2 periosteal cells were harvested from the P3 bones of C57BL/6 mice at 8 weeks old, as previously described (Jaramillo et al., 2023). In brief, bones were placed in a 12-well culture plate and allowed to expand through repeated replating for final culture in a 100-mm plate. Cells were then trypsinized, seeded at 20 K cells/well in a 24-well Agilent Seahorse plate (Agilent, Santa Clara, CA), and evaluated the following day using the Seahorse Mito Stress Test (Agilent, Santa Clara, CA) using the standard protocol and 0.75 μM FCCP concentration (*n* = 10 wells per group).

### Single-cell RNAseq analysis

Previously published scRNAseq datasets obtained from days 10 and 14 post-amputation of the P3 or P2 digit were downloaded from the Gene Expression Omnibus repository and analyzed in *Seurat*. Cell clusters were identified using standard marker genes as we have previously published (Tower et al., 2022). To calculate senescent transcriptional profiles, the *Addmodulescore* function was used in combination with gene lists derived from the previously published “SenMayo” annotations (Saul et al., 2022).

## Data Availability

The RNAseq data set accession numbers can be found in the referenced works.

## Supplementary material

SUPPLEMENTARY FIGURE S1 Slice culture of unamputated **(A)** and P3 digit at 10 days after injury **(B)** stained for SA-βGal at pH 4 as a control X-gal activity shows majority of cells stained blue. The white arrow indicates the marrow cavity.

SUPPLEMENTARY FIGURE S2 Immunofluorescent staining for p21 (Waf1/Cip) in tissue of injured digits. Lateral sections of the injured digit at D10. p21 stains the nuclei of positive cells (white arrows) scattered around the injured bone in both P3 and P2 injuries. The yellow channel indicates the autofluorescent background noise. Representative images of n = 3 animals. p21 (green), background (yellow).

SUPPLEMENTARY FIGURE S3 P2- and P3-derived cells express differential senescent phenotypes. **(A)** Modular scores for gene expression using the SenNet profile are quantified to compare P2 and P3 injuries across all cells using scRNAseq datasets reanalyzed from previous publications (Johnson et al., 2020; Storer et al., 2020). Module score of the complete list (SenNet) based off of previously published datasets (Suryadevara et al., 2024). **(B)** Modular scores for gene expression using the SenMayo profile are quantified to compare P2 and P3 injuries across all cells. Module score of the complete list (SenMayo) based off of previously published datasets (Saul et al., 2022). **(C)** Modular scores for gene expression for the SenMayo profile are quantified to compare P2 and P3 injuries in cluster 1 (mesenchymal cells). Genes grouped by following categories: lysosomal, growth factors, matrix metalloproteases (MMPs), cytokines and chemokines, transmembrane proteins, protease inhibitors, protein modifying enzymes, transcription factors and regulators (TF+Reg), intracellular signaling (ICell Sig), and miscellaneous (Misc).

SUPPLEMENTARY TABLE S1 Full panel of genes used for modular scoring of the *in vivo* cellular senescent profile.
