# Supplementary material for: Osteoprogenitor cells from non-regenerative bone show greater resistance to cellular stress than those from regenerative bone
Source: Front Cell Dev Biol. 2025 Dec 18;13:1684670. doi: 10.3389/fcell.2025.1684670 (PMC12756367; doi:10.3389/fcell.2025.1684670)
Supplement: Supplementary file 1 [file Presentation1.pptx]

## Slide 1
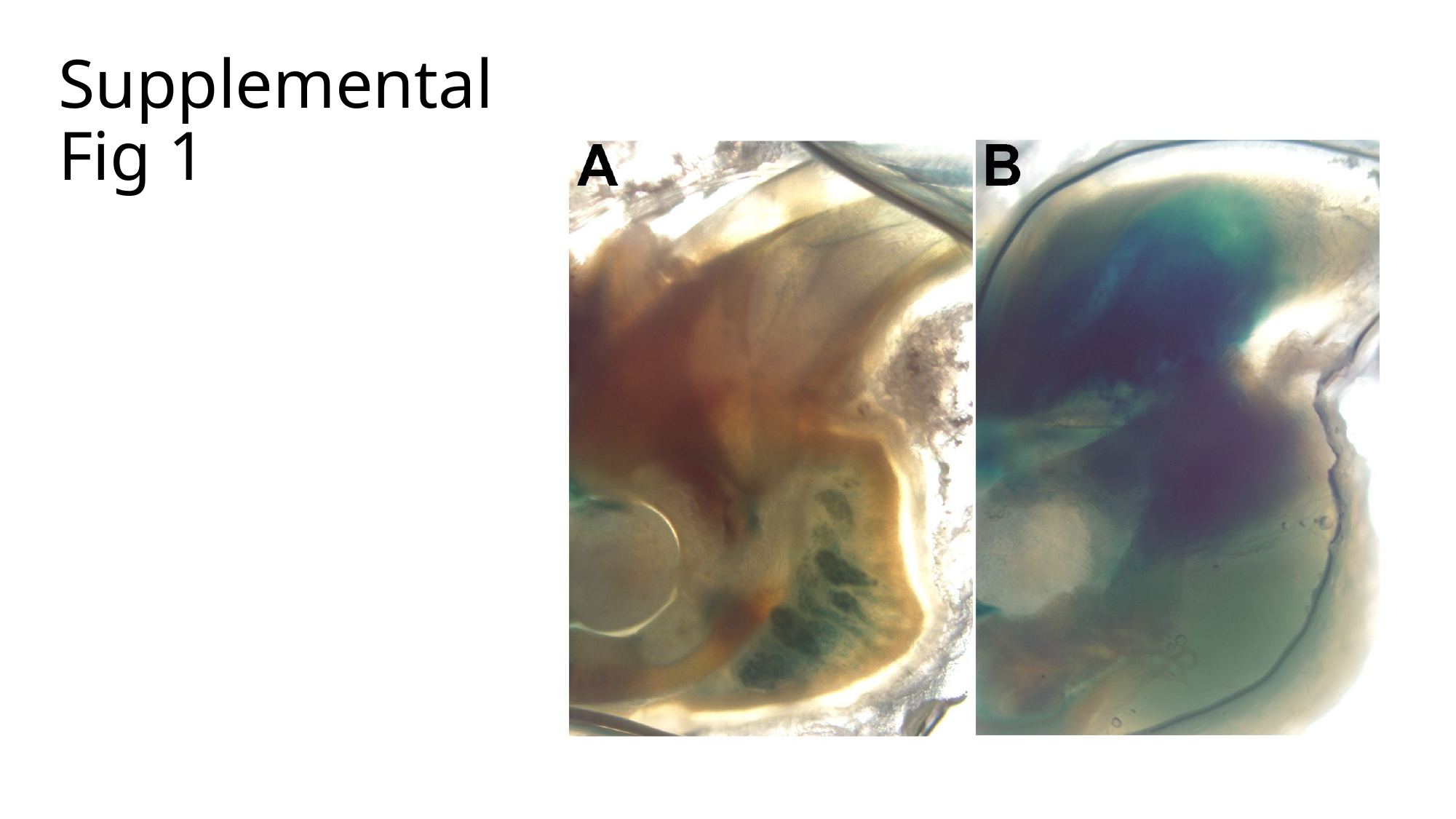

# Supplemental Fig 1

## Slide 2
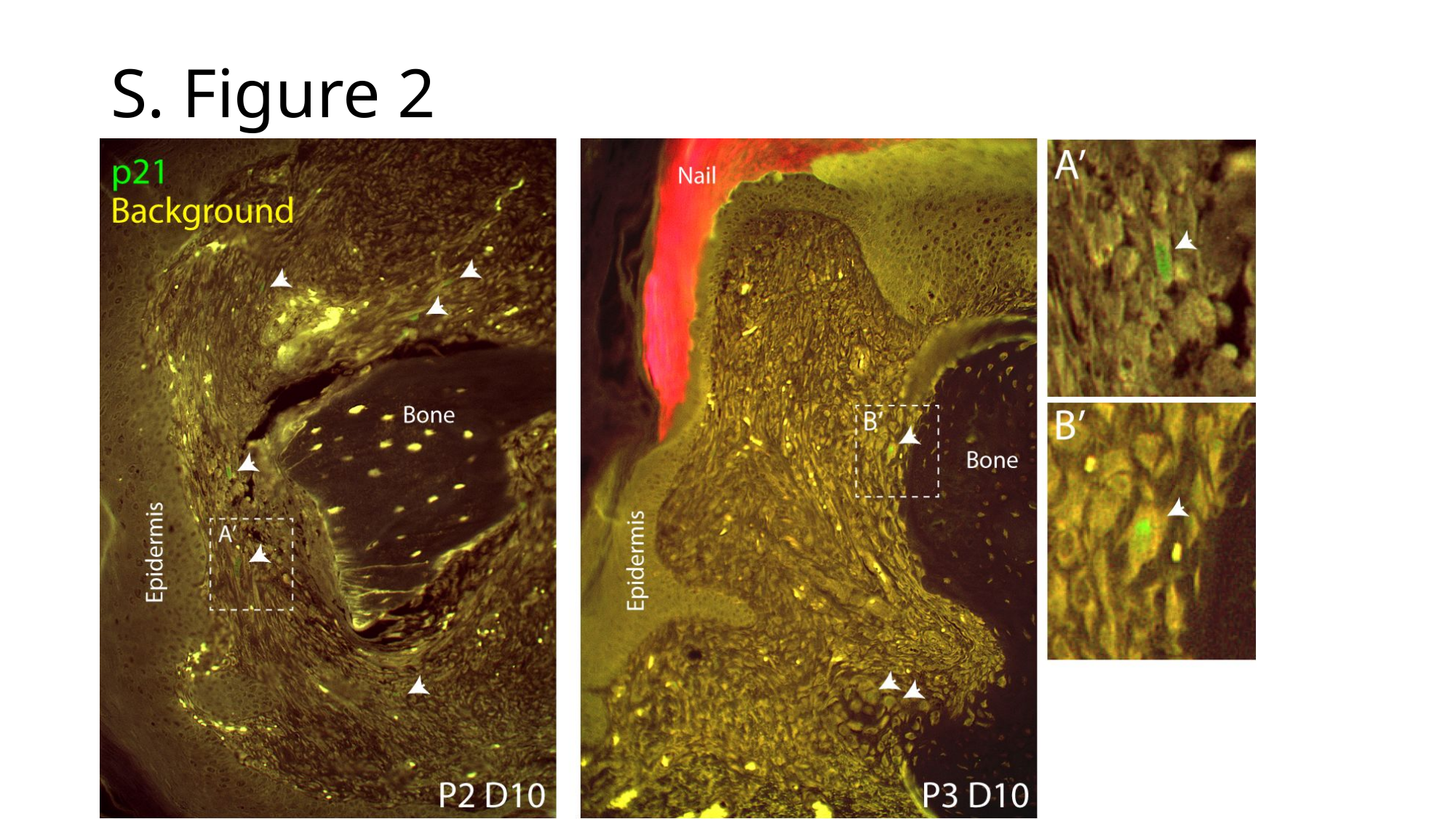

# S. Figure 2

## Slide 3
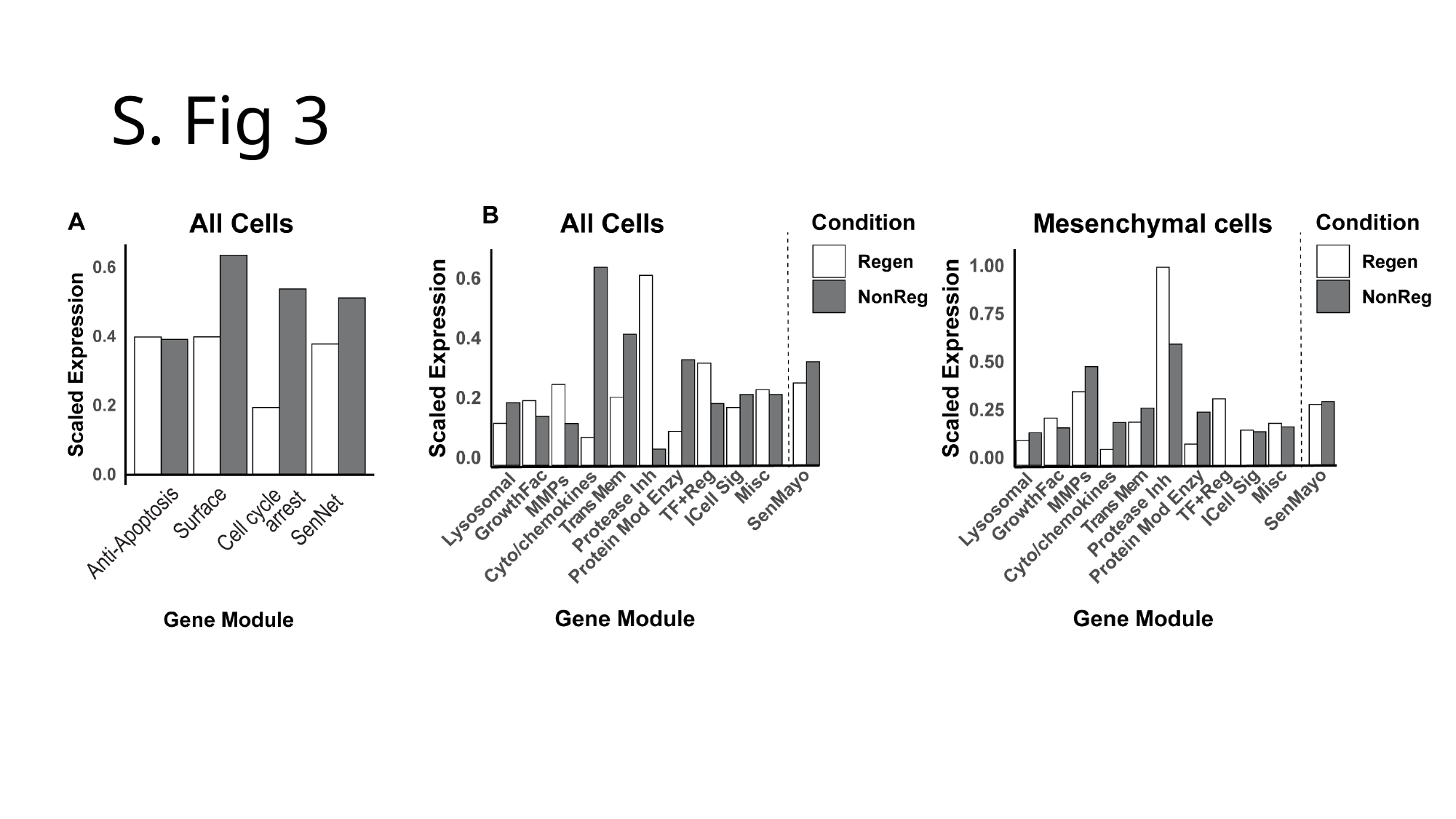

# S. Fig 3

## Slide 4
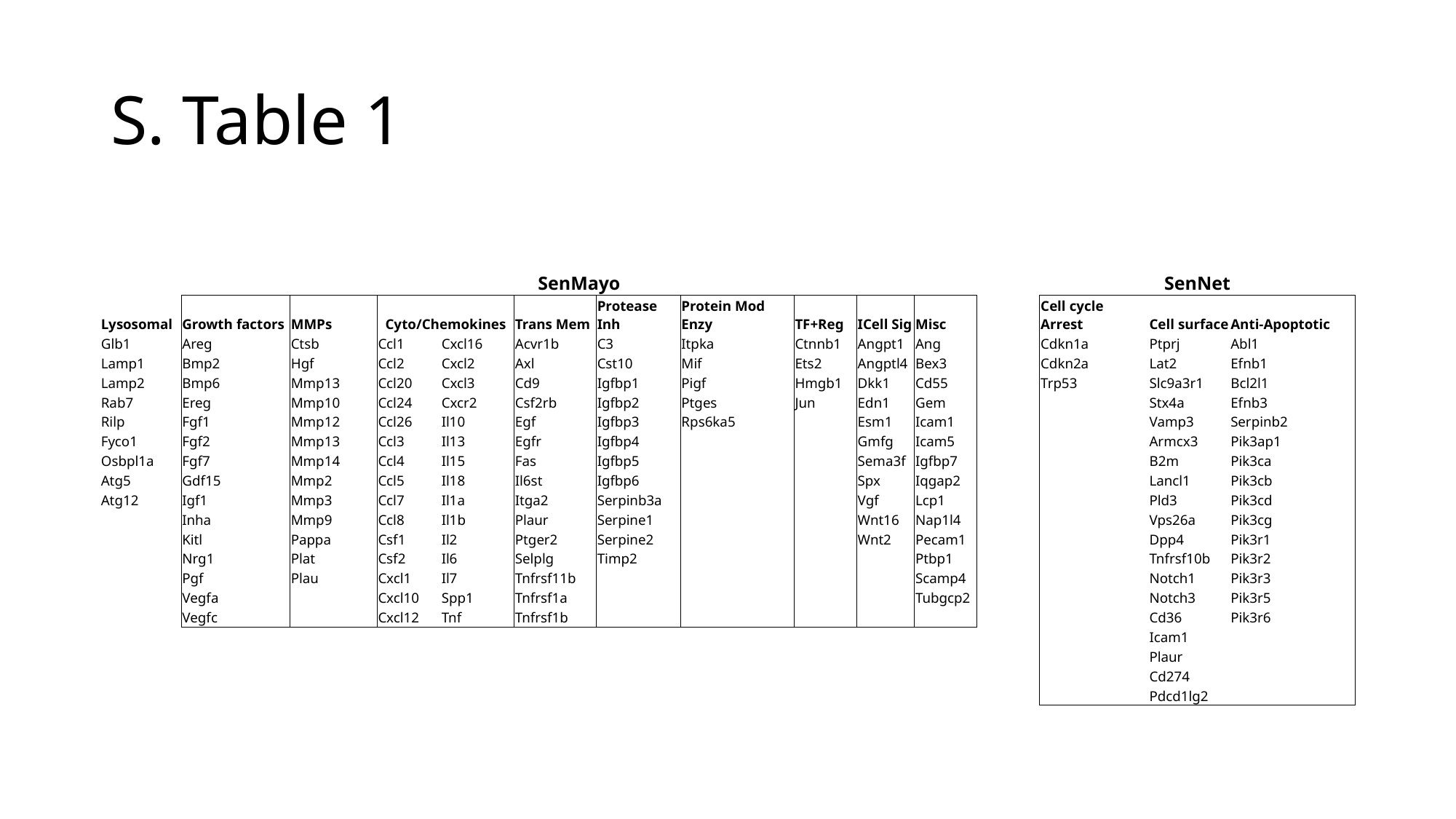

# S. Table 1
| | SenMayo | | | | | | | | | | | SenNet | | | |
| --- | --- | --- | --- | --- | --- | --- | --- | --- | --- | --- | --- | --- | --- | --- | --- |
| Lysosomal | Growth factors | MMPs | Cyto/Chemokines | | Trans Mem | Protease Inh | Protein Mod Enzy | TF+Reg | ICell Sig | Misc | | Cell cycle Arrest | Cell surface | Anti-Apoptotic | |
| Glb1 | Areg | Ctsb | Ccl1 | Cxcl16 | Acvr1b | C3 | Itpka | Ctnnb1 | Angpt1 | Ang | | Cdkn1a | Ptprj | Abl1 | |
| Lamp1 | Bmp2 | Hgf | Ccl2 | Cxcl2 | Axl | Cst10 | Mif | Ets2 | Angptl4 | Bex3 | | Cdkn2a | Lat2 | Efnb1 | |
| Lamp2 | Bmp6 | Mmp13 | Ccl20 | Cxcl3 | Cd9 | Igfbp1 | Pigf | Hmgb1 | Dkk1 | Cd55 | | Trp53 | Slc9a3r1 | Bcl2l1 | |
| Rab7 | Ereg | Mmp10 | Ccl24 | Cxcr2 | Csf2rb | Igfbp2 | Ptges | Jun | Edn1 | Gem | | | Stx4a | Efnb3 | |
| Rilp | Fgf1 | Mmp12 | Ccl26 | Il10 | Egf | Igfbp3 | Rps6ka5 | | Esm1 | Icam1 | | | Vamp3 | Serpinb2 | |
| Fyco1 | Fgf2 | Mmp13 | Ccl3 | Il13 | Egfr | Igfbp4 | | | Gmfg | Icam5 | | | Armcx3 | Pik3ap1 | |
| Osbpl1a | Fgf7 | Mmp14 | Ccl4 | Il15 | Fas | Igfbp5 | | | Sema3f | Igfbp7 | | | B2m | Pik3ca | |
| Atg5 | Gdf15 | Mmp2 | Ccl5 | Il18 | Il6st | Igfbp6 | | | Spx | Iqgap2 | | | Lancl1 | Pik3cb | |
| Atg12 | Igf1 | Mmp3 | Ccl7 | Il1a | Itga2 | Serpinb3a | | | Vgf | Lcp1 | | | Pld3 | Pik3cd | |
| | Inha | Mmp9 | Ccl8 | Il1b | Plaur | Serpine1 | | | Wnt16 | Nap1l4 | | | Vps26a | Pik3cg | |
| | Kitl | Pappa | Csf1 | Il2 | Ptger2 | Serpine2 | | | Wnt2 | Pecam1 | | | Dpp4 | Pik3r1 | |
| | Nrg1 | Plat | Csf2 | Il6 | Selplg | Timp2 | | | | Ptbp1 | | | Tnfrsf10b | Pik3r2 | |
| | Pgf | Plau | Cxcl1 | Il7 | Tnfrsf11b | | | | | Scamp4 | | | Notch1 | Pik3r3 | |
| | Vegfa | | Cxcl10 | Spp1 | Tnfrsf1a | | | | | Tubgcp2 | | | Notch3 | Pik3r5 | |
| | Vegfc | | Cxcl12 | Tnf | Tnfrsf1b | | | | | | | | Cd36 | Pik3r6 | |
| | | | | | | | | | | | | | Icam1 | | |
| | | | | | | | | | | | | | Plaur | | |
| | | | | | | | | | | | | | Cd274 | | |
| | | | | | | | | | | | | | Pdcd1lg2 | | |
